# Cutting-Edge HEK293T Protein-Integrated Lipid Nanostructures: Boosting Biocompatibility and Efficacy

**DOI:** 10.3390/ijms25063294

**Published:** 2024-03-14

**Authors:** Jung-Hyun Park, Cheng-Zhe Bai, Jeong-Hun Kwak, Ho-Joong Choi, Dosang Lee, Ha-Eun Hong, Ok-Hee Kim, Say-June Kim

**Affiliations:** 1 Department of Surgery, Eunpyeong St. Mary’s Hospital, College of Medicine, The Catholic University of Korea, Seoul 06591, Republic of Korea; 2 Catholic Central Laboratory of Surgery, College of Medicine, The Catholic University of Korea, Seoul 06591, Republic of Korea; 3 Translational Research Team, Surginex Co., Ltd., Seoul 06591, Republic of Korea; 4 Department of Surgery, Seoul St. Mary’s Hospital, College of Medicine, The Catholic University of Korea, 222 Banpo-daero, Seocho-gu, Seoul 06591, Republic of Korea

**Keywords:** anticancer therapeutics, artificial exosomes, HEK293T cells, lipid nanostructures, membrane proteins

## Abstract

Recently, artificial exosomes have been developed to overcome the challenges of natural exosomes, such as production scalability and stability. In the production of artificial exosomes, the incorporation of membrane proteins into lipid nanostructures is emerging as a notable approach for enhancing biocompatibility and treatment efficacy. This study focuses on incorporating HEK293T cell-derived membrane proteins into liposomes to create membrane-protein-bound liposomes (MPLCs), with the goal of improving their effectiveness as anticancer therapeutics. MPLCs were generated by combining two key elements: lipid components that are identical to those in conventional liposomes (CLs) and membrane protein components uniquely derived from HEK293T cells. An extensive comparison of CLs and MPLCs was conducted across multiple in vitro and in vivo cancer models, employing advanced techniques such as cryo-TEM (tramsmission electron microscopy) imaging and FT-IR (fourier transform infrared spectroscopy). MPLCs displayed superior membrane fusion capabilities in cancer cell lines, with significantly higher cellular uptake. Additionally, MPLCs maintained their morphology and size better than CLs when exposed to FBS (fetal bovine serum), suggesting enhanced serum stability. In a xenograft mouse model using HeLa and ASPC cancer cells, intravenous administration of MPLCs MPLCs accumulated more in tumor tissues, highlighting their potential for targeted cancer therapy. Overall, these results indicate that MPLCs have superior tumor-targeting properties, possibly attributable to their membrane protein composition, offering promising prospects for enhancing drug delivery efficiency in cancer treatments. This research could offer new clinical application opportunities, as it uses MPLCs with membrane proteins from HEK293T cells, which are known for their efficient production and compatibility with GMP (good manufacturing practice) standards.

## 1. Introduction

The necessity for artificial exosomes arises from their potential as powerful nanocarriers in drug delivery systems, where they address significant challenges associated with natural exosomes, such as scalability of production, efficient isolation, and stability concerns [1]. These synthetic counterparts are designed to leverage the intrinsic benefits of natural exosomes while mitigating their limitations. Specifically, in targeted cancer therapy, artificial exosomes hold great promise by merging the beneficial features of natural exosomes, such as biocompatibility and targeted delivery, with the strength, ease of customization, and production scalability of synthetic nanoparticles [2,3]. This hybrid approach aims to enhance the efficacy and precision of drug delivery, making artificial exosomes a crucial development in the advancement of nanomedicine.

Bottom–up strategies are one of the most favored ways of generating artificial exosomes [1,4,5,6]. This approach involves starting with molecular or atomic components to construct more complex structures through a stepwise assembly process [1]. This method is highlighted by the use of liposomes, lipid-based nanoparticles that emulate the bilayer structure of cell membranes and are analogous to natural exosomes in form and function. Research in this area is diverse, exploring various materials and methods to fabricate artificial exosomes [7,8,9,10]. Studies have delved into optimizing liposome formulations to enhance their resemblance to natural exosomes, experimenting with different lipid compositions, surface modifications, and encapsulation techniques [8,9,10]. This approach not only provides insights into the essential properties of exosomes but also guides the development of more effective and tailored nanocarriers for targeted therapeutic applications.

Incorporating membrane proteins into lipid nanostructures has become a significant area of research in nanomedicine, with the aim of increasing biocompatibility and treatment efficacy [1]. Numerous studies have investigated this for anti-tumor therapy, focusing on proteins derived from tumor cells, immune cells, or red blood cells (RBCs) due to their anticipated targetability towards tumor cells [10,11,12,13,14,15]. In our study, we utilized membrane proteins from HEK293T cells, creating membrane-protein-bound liposomes (MPLCs). This approach assumes that even non-tumor-specific membrane proteins can achieve high biocompatibility, targetability, and efficacy when properly combined with liposomes. Crucially, HEK293T cells, known for their ease of growth, transfection amenability, and human origin, offer numerous advantages in membrane protein production, including scalability and regulatory compliance [16,17]. 

In this research, our objective was to create artificial exosomes, termed membrane-protein-bound lipid complex, MPLCs in short, utilizing membrane proteins from HEK293T cells. This endeavor was followed by comprehensive studies to assess the effectiveness of MPLCs against conventional liposomes (CLs). These studies encompassed a range of assays, including those evaluating immunogenicity, biocompatibility, intracellular delivery, and in vivo targetability. The demonstrated efficacy of MPLCs over CLs suggests a promising new direction in treatment modalities. This is particularly significant given the industrial applicability of HEK293T cells, known for their high productivity, ease of handling, and suitability for Current Good Manufacturing Practice (cGMP) standards.

## 2. Results

### 2.1. Development of Membrane-Protein-Bound Lipid Complexes (MPLCs)

**Figure 1:**
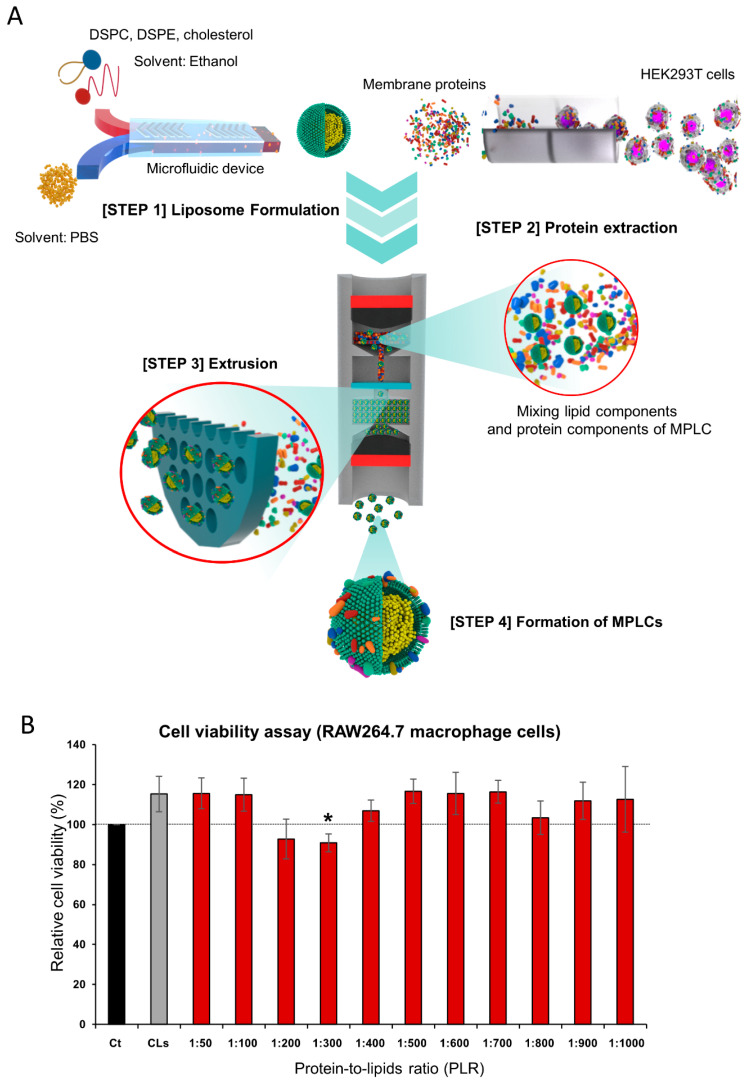
Development of membrane-protein-bound lipid complexes (MPLCs). (**A**) Schematic illustration of full process of generating MPLCs. It begins with the extraction of membrane proteins from HEK293T cells, followed by their combination with a lipid mixture (DSPC, DSPE, and cholesterol) through a microfluidic method. The mixture then undergoes a 15-cycle extrusion process using a 100 nm pore-sized cellulose acetate membrane, resulting in the formation of MPLCs. (**B**) Cell viability assay for the determination of optimal protein-to-lipid ratio (PLR). Different PLRs ranging from 1:50 to 1:10,000 were tested using RAW264.7 macrophage cells. The assay results indicate that a PLR of 1:300 results in the lowest cell viability, suggesting the highest biocompatibility for MPLCs. Values are presented as mean ± standard deviation of three independent experiments. * *p* < 0.05.

In this research, the focus was on developing and analyzing MPLCs, which are artificial exosomes produced by integrating membrane proteins from HEK293T cells with CLs (Figure 1A). The synthesis of CLs was achieved through advanced microfluidic methods. This process involved the combination of distinct lipid molecules: DSPC, DSPE, and cholesterol. By contrast, MPLCs consist of two essential components: lipid components and membrane protein components. The former precisely matches those found in the CLs, ensuring a comparable baseline for comparison, and the latter is uniquely sourced from HEK293T cells. The process of creating MPLCs is as follows. Initially, membrane proteins were obtained through a process of permeabilization and solubilization of the HEK293T cells. Once these membrane proteins were prepared, they were combined with the pre-formed lipid components by extrusion process. 

The protein-to-lipid ratio (PLR) in MPLCs was established by comparing the weight of the protein to that of DSPC in micrograms. A range of PLRs for MPLCs, namely 1 to 50, 1 to 100, and up to 1 to 10,000, were prepared to determine the most effective composition. These varying PLRs of MPLCs were evaluated using a cell viability assay with RAW264.7 macrophage cells. In this context, reduced cell viability is indicative of lower immunogenicity and higher biocompatibility. The results showed that a PLR of 1:300 led to the lowest cell viability, suggesting optimal biocompatibility (Figure 1B). Consequently, in accordance with the established PLR of 1:300 for our study, MPLCs conforming to this ratio were prepared by mixing 33.3 μg of protein with 10 mg of lipid.

### 2.2. Characterization of MPLCs: Morphology, Composition, and Thermal Properties

**Figure 2:**
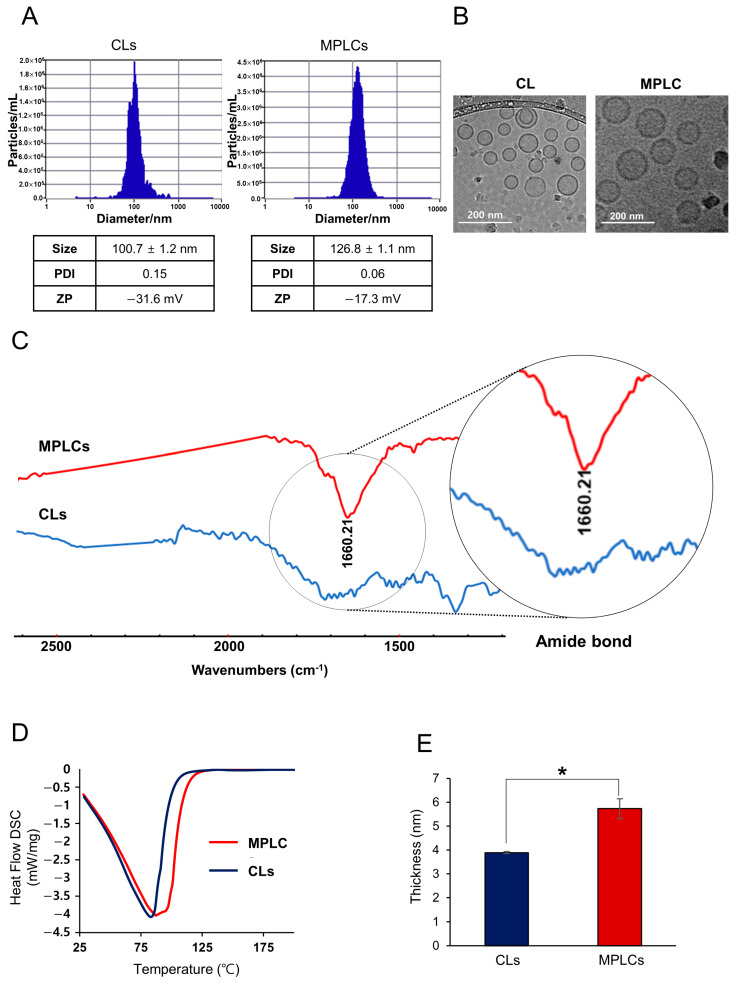
Determination of physical characteristics of MPLCs. (**A**) ZetavVew analysis of nanoparticles. MPLCs have a larger average diameter, lower PDI, and reduced zeta potential compared to CLs, suggesting enhanced stability and potential for more precise targeting. (**B**) Morphological comparison using cryo-TEM. CLs exhibit a uniform spherical shape with well-defined bilayers, whereas MPLCs display a diverse morphology with surface-bound proteins, indicating improved cellular interaction capabilities. (**C**) Fourier Transform Infrared (FT-IR) spectroscopy, highlighting the amide bond peak in MPLCs absent in CLs. This peak indicates the presence of proteins in MPLCs, distinguishing them from the protein-free lipid profile of CLs. (**D**) Differential Scanning Calorimetry (DSC) demonstrating thermal properties of nanoparticles. MPLCs exhibit a unique endothermic peak, suggesting altered thermotropic behavior of their lipid bilayers, potentially impacting membrane fluidity and stability. (**E**) Comparison of membrane thickness. Cryo-TEM images, analyzed with ImageJ, reveal a significant increase in membrane thickness in MPLCs compared to CLs. This increased thickness supports the successful integration of proteins into the MPLC structure. Values are presented as mean ± standard deviation of three independent experiments. * *p* < 0.05.

ZetaView analysis demonstrated that conventional liposomes (CL) exhibited an average diameter of 100.7 ± 1.2 nm, a polydispersity index (PDI) of 0.15, and a zeta potential of 31.6 mV. In contrast, the MPLCs presented an average diameter of 126.8 ± 1.1 nm, a lower PDI of 0.06, and a reduced zeta potential of −17.3 mV (Figure 2A). The observed variations, characterized by MPLCs’ increased size, reduced PDI, and higher zeta potential, imply enhanced stability and possibly more precise targeting abilities. This can be attributed to the integral membrane proteins, which are likely to impact the interactions between liposomes and cells, thereby potentially optimizing the efficacy of drug delivery. The cryo-TEM image distinctly shows that CLs possess a uniform spherical morphology and well-defined bilayers, whereas MPLCs are characterized by a diverse morphology with apparent surface-bound proteins, which may confer improved cellular interaction capabilities (Figure 2B).

FT-IR spectroscopy is performed to identify and quantify molecular components within a sample by detecting their unique vibrational modes, offering detailed insights into molecular structure and composition. In the FT-IR spectrum, the MPLCs exhibit a prominent amide bond peak around 1600–1700 cm^−1^, indicative of protein presence, which is absent in the CLs spectrum. This difference underscores the successful incorporation of proteinaceous components within the MPLCs, contrasting with the protein-free lipid profile of CLs.

Differential Scanning Calorimetry (DSC) was employed to compare the thermal properties of CLs and MPLCs. The DSC curve demonstrates that MPLCs exhibit a distinct thermal transition, with a pronounced endothermic peak, compared to CLs, suggesting differences in the thermotropic behavior of their lipid bilayers (Figure 2D). This shift in the MPLCs’ heat flow profile indicates a potential alteration in membrane fluidity and stability, likely due to the interaction of membrane proteins with the lipid components. The comparison reveals that protein incorporation in MPLCs significantly influences their thermal response, which could have implications for their functionality as drug delivery vehicles. Subsequently, cryo-TEM imaging and subsequent analysis with ImageJ v.1.53k revealed that MPLCs exhibit a notably increased membrane thickness in comparison to CLs (Figure 2E). The enhanced membrane thickness substantiates the integration of proteins into the MPLC architecture, confirming their successful synthesis.

### 2.3. Immunogenicity and Biocompatibility of MPLCs

**Figure 3:**
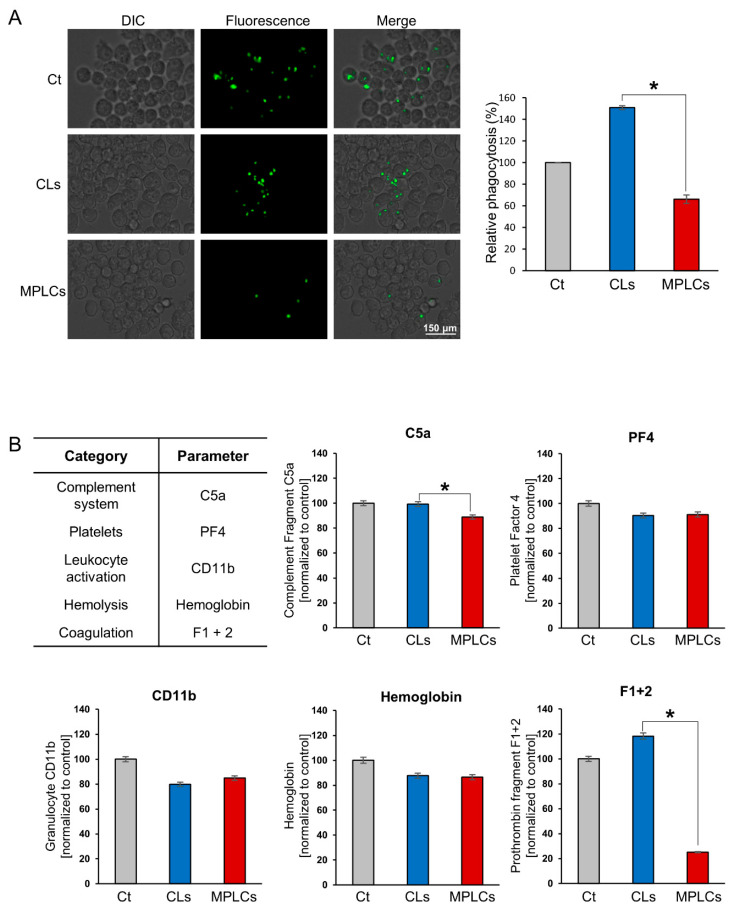
Immunogenicity and biocompatibility of MPLCs. (**A**) Phagocytosis assay, assessing the immunogenicity of MPLCs in comparison to CLs. Green fluorescence indicates the uptake of zymosan particles by immune cells in the assay. MPLCs exhibit a lower phagocytosis rate in macrophages than CLs, implying reduced immunogenicity, which is favorable for applications requiring minimal immune response. (**B**) Hemocompatibility analysis. Human RBCs were incubated with CLs and MPLCs at 37 °C for 2 h, followed by centrifugation to separate the serum for analysis using ELISA kit. MPLCs had significantly lower levels of C5a (a component of the complement system) and F1 + 2 (Prothrombin Fragment 1 + 2, indicative of prothrombin breakdown), indicating better biocompatibility (*p* < 0.05). Other biomarkers like PF4, CD11b, and hemoglobin release were similar between MPLCs and CLs. Values are presented as mean ± standard deviation of three independent experiments. * *p* < 0.05.

A phagocytosis assay employing a pre-labeled zymosan particle was conducted to measure the phagocytosis rate following the treatment of immune cells with CLs and MPLCs, respectively. The assay utilized green fluorescence to track the uptake of zymosan particles by the cells, with a higher incidence of phagocytosis correlating to increased fluorescence. The results indicate that the MPLCs elicited the lowest phagocytosis rate in macrophages compared to CLs (Figure 3A). This suggests that MPLCs may exhibit lower immunogenicity than CLs, potentially indicating their suitability for applications where reduced immune response is desirable. 

In the investigation of hemocompatibility, the analysis focused on the interactions of human red blood cells (RBCs) with CLs and MPLCs, respectively. Human RBCs were incubated with CLs and MPLCs, respectively, at 37 °C for 2 h, after which centrifugation was utilized to isolate the serum for further analysis using an ELISA kit. The investigation revealed that MPLCs exhibited significantly reduced concentrations of C5a (a component of the complement system) and F1 + 2 (Prothrombin Fragment 1 + 2, indicative of prothrombin breakdown), evidencing superior biocompatibility (*p* < 0.05) (Figure 3B). Other measured biomarkers, including PF4 (Platelet Factor 4, a marker of platelet activation), CD11b (a marker of leukocyte activation), and hemoglobin release, did not demonstrate marked disparities between the MPLC and CL groups. 

### 2.4. Enhanced In Vitro Intracellular Delivery and Serum Stability of MPLCs

**Figure 4:**
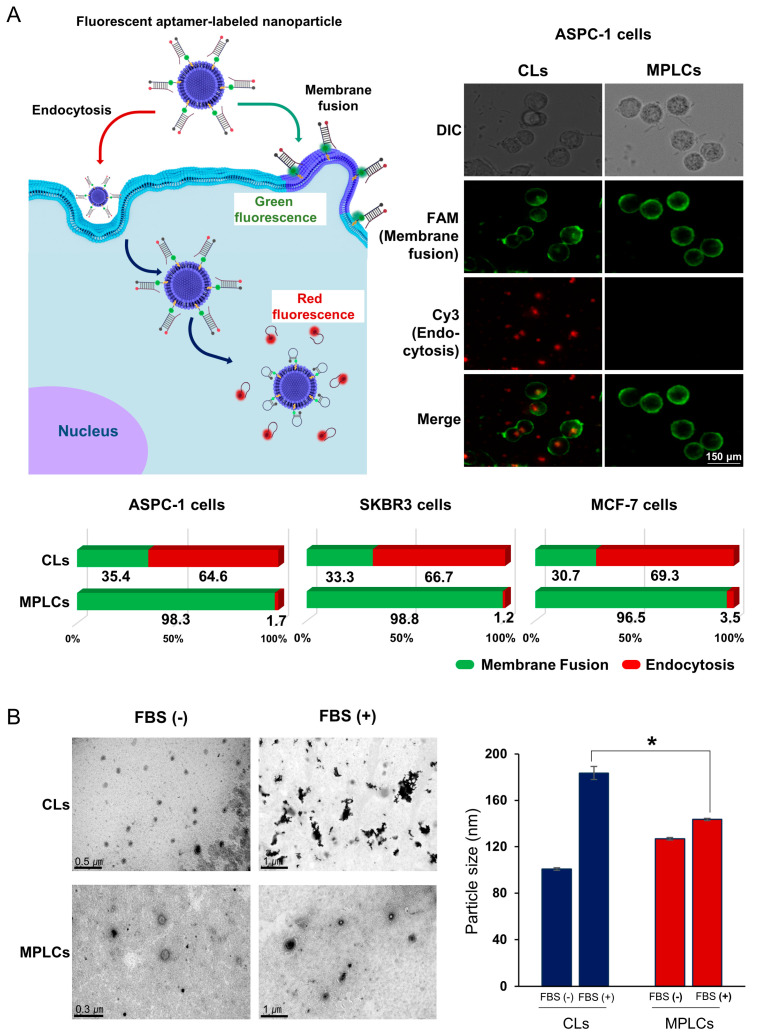
Enhanced intracellular delivery and serum stability of MPLCs. (**A**) Assessment of intracellular delivery mechanisms through fluorescence microscopy. Each cancer cell line was treated with aptamer-incorporated CLs and MPLCs, respectively, and the subsequent uptake was measured by fluorescence microscopy. Aptamers were dual-labeled with Cy3 dye, which emits red fluorescence for endocytosis tracking, and with FAM dye, emitting green fluorescence for membrane fusion tracking (**left**). (**A**) (**right**) presents representative fluorescence microscopy images of ASPC1 cells following treatment with aptamer-labeled nanoparticles. Following incubation with ASPC1, SKBR3, and MCF-7 cancer cell lines, fluorescence microscopy analysis showed that MPLCs significantly favored fusion-mediated internalization over CLs in all cell lines, with notably higher fusion rates (**bottom**). (**B**) Evaluation of nanoparticle stability in fetal bovine serum (FBS), a simulated in vivo environment. TEM images demonstrate that, unlike CLs, MPLCs preserved their structural integrity and dimensional uniformity, suggesting enhanced serum stability, likely due to their protein coating. ZetaView analysis showed a significant increase in particle size for CLs after FBS treatment (*p* < 0.05), while MPLCs maintained consistent particle sizes, indicating their stability. Values are presented as mean ± standard deviation of three independent experiments. * *p* < 0.05.

For the determination of intracellular delivery mechanisms of CLs or MPLCs, aptamers were labeled on the nanoparticles, with one sequence modified with Cy3 dye to signal endocytosis through red fluorescence and a complementary sequence tagged with FAM dye to indicate membrane fusion through green fluorescence (Figure 4A). Aptamer-labeled CLs and MPLCs, respectively, were incubated with ASPC1 pancreatic, SKBR3, and MCF-7 breast cancer cell lines. After a three-hour treatment, intracellular uptake was analyzed via fluorescence microscopy. The analysis demonstrated that MPLCs favored fusion-mediated internalization significantly more than CLs across all cell lines tested. In the case of ASPC1, SKBR3, and MCF-7 cells, MPLCs displayed fusion rates of 98.3%, 98.8%, and 96.5%, respectively, in stark contrast to the CLs, which exhibited fusion rates of 35.2%, 33.3%, and 30.7%. This stark difference highlights the superior membrane fusion capabilities of MPLCs compared to CLs, suggesting enhanced intracellular delivery potential.

Subsequently, the stability of the nanoparticles was evaluated by exposing each nanoparticle—CLs and MPLCs—to fetal bovine serum (FBS), which mimics the in vivo environment. The nanoparticles were treated with 0.1% FBS solution at 37 °C for 2 h, dialyzed to remove unreacted FBS, and then analyzed for morphological changes via TEM and for size alterations using ZetaView. Post-FBS treatment, CLs exhibited significant aggregation and morphological alterations, evidenced by increased particle size and changed morphology in TEM images. In contrast, MPLCs showed minimal aggregation and maintained their morphology and size, suggesting that the protein coating on MPLCs enhances their stability in biologically relevant conditions (Figure 4B left). In the comparison of particle sizes before and after FBS treatment using ZetaView, CLs demonstrated a significant increase in particle size post-FBS exposure (*p* < 0.05), whereas MPLCs did not exhibit a statistically meaningful increment in particle size (Figure 4B right).

### 2.5. Enhanced Cellular Uptake of MPLCs in Cancer Cell Lines

**Figure 5:**
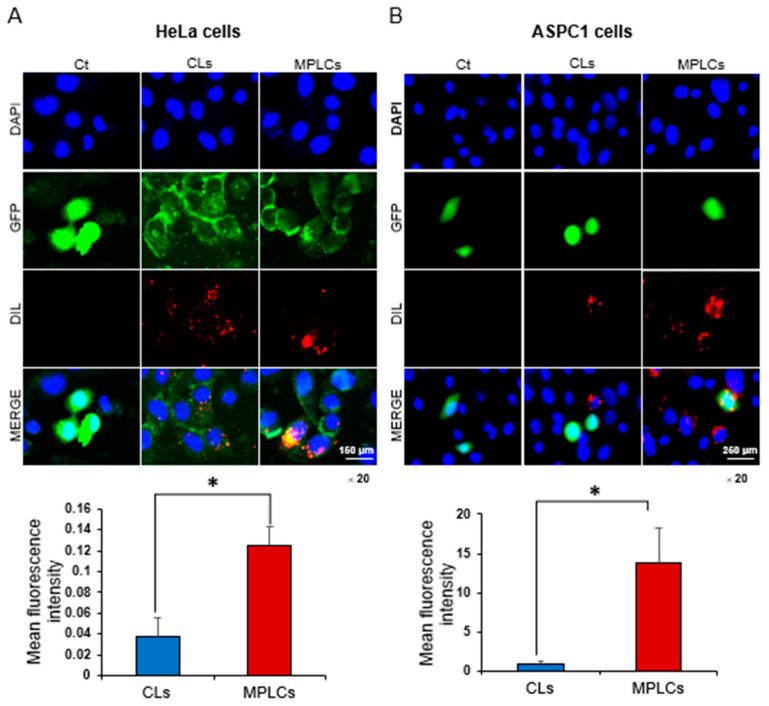
Enhanced cellular uptake of MPLCs in cancer cell lines. (**A**) Cellular uptake in HeLa cervical cancer cells. Fluorescence micrographs were acquired following the treatment of HeLa cells with DiL-labeled CLs and MPLCs, respectively. The HeLa cells, transfected with green fluorescent protein (GFP), demonstrated significantly higher uptake of MPLCs (*p* < 0.05) than CLs, evidenced by the intense red fluorescence from the DiL dye, indicating enhanced internalization. (**B**) Cellular uptake in ASPC1 pancreatic cancer cells. This panel shows uptake patterns consistent with those observed in HeLa cervical cancer cells. Values are presented as mean ± standard deviation of three independent experiments. * *p* < 0.05.

In assessing the efficiency of cellular uptake, HeLa cervical cancer cells and ASPC1 pancreatic cancer cells were subjected to treatments with CLs and MPLCs, respectively. Prior to treatment, cells were transfected with green fluorescent protein (GFP), serving as a marker for transfection efficiency. Following transfection, cells were exposed to DiL-labeled CLs or MPLCs to track their cellular uptake. The MPLCs demonstrated a significantly higher uptake in both cell lines, as indicated by the intensified red fluorescence from the DiL dye, which was also quantitatively corroborated by the analysis of fluorescence intensity values (Figure 5A,B). These findings not only highlight the superior internalization of MPLCs but also suggest their promising capabilities in enhancing the delivery of therapeutics into target cells. The increased uptake of MPLCs may be attributed to the presence of membrane proteins, which potentially facilitate the recognition and subsequent internalization by the cancer cells.

### 2.6. Determination of In Vivo Targetability and Intracellular Delivery of MPLCs

**Figure 6:**
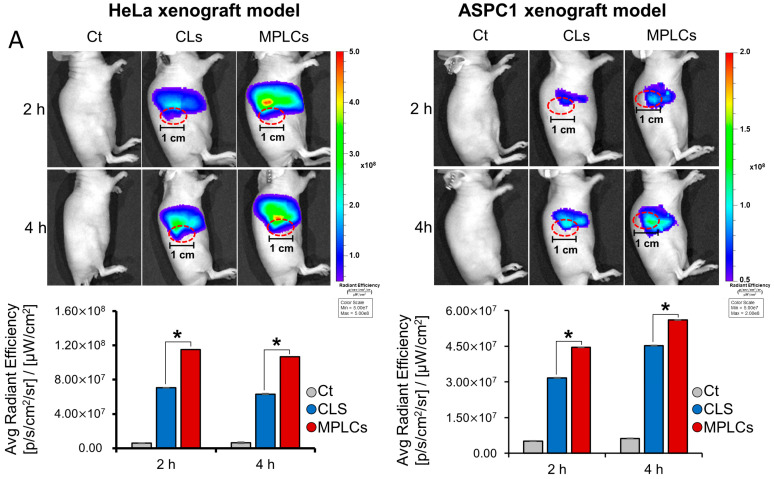

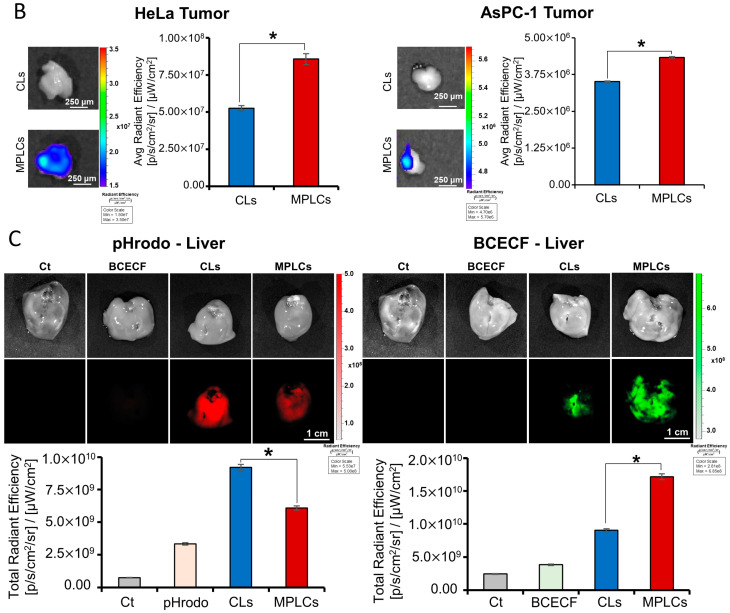
In vivo determination of targetability and intracellular delivery of MPLCs. (**A**) Sequential whole-body fluorescence imaging after individual treatments. BALB/c nude mice bearing HeLa (**left**) and ASPC1 (**right**) cell xenografts received administrations of DiR-labeled CLs and MPLCs, respectively. MPLCs demonstrated a higher accumulation in near-tumor areas compared to CLs, signifying enhanced tumor-targeting efficacy. The tumor region was delineated with red dotted lines. (**B**) Fluorescence imaging of the excised tumor tissues from the xenograft mice. MPLCs showed a pronounced preferential accumulation in tumor tissue over CLs. (**C**) Assessment of intracellular delivery mechanisms through nanoparticle labeling. Mice received injections of nanoparticles (either CLs or MPLCs) tagged with pHrodo or BCECF dyes. In environments like the lysosome, where the pH is acidic (around pH 4–5), pHrodo dye exhibits red fluorescence, whereas BCECF dye emits green fluorescence in other conditions. Subsequent fluorescence microscopic analysis of the excised livers indicated that CLs predominantly displayed red fluorescence, suggesting endocytosis, whereas MPLCs predominantly showed green fluorescence, signifying uptake predominantly via membrane fusion. * *p* < 0.05.

In a xenograft mouse model experiment, the in vivo targetability of CLs and MPLCs was evaluated to assess their potential for targeted cancer therapy. For xenograft model creation, 5 × 10^6^ cells of both HeLa and ASPC1 cell lines were injected into the flanks of BALB/c nude mice. DiR-labeled nanoparticles were prepared by incubating the respective nanoparticles with 10 µg/mL DiR at 37 °C for 20 min. Mice with HeLa and ASPC1 xenografts were given a tail vein injection of 10^9^ particles in 100 µL, one and two weeks post-engraftment, respectively. Sequential whole-body fluorescence imaging conducted at 2 and 4 h intervals post-injection revealed that MPLCs achieved significantly higher accumulation in the near-tumor areas compared to CLs (Figure 6A). The fluorescence distribution of CLs and MPLCs in major organs of mouse xenograft models is detailed in Supplementary Figure S1, indicating preferential accumulation in the liver and spleen. Additionally, when comparing the two treatment groups, MPLCs showed a superior average radiant efficiency in all organs assessed (heart, lung, liver, kidney, spleen, and pancreas) versus their CL counterparts. The targeted delivery was further substantiated by the fluorescence imaging of excised tumor tissues, indicating a marked preference for MPLCs for tumor tissue over CLs (Figure 6B). These findings suggest MPLCs possess enhanced tumor-targeting capabilities, likely due to their membrane protein constituents, which could be leveraged to improve the efficacy of drug delivery in oncological applications.

Finally, an additional in vivo experiment was conducted to ascertain the intracellular delivery mechanisms of CLs and MPLCs. The nanoparticles (CLs and MPLCs) were stained with either pHrodo or BCECF dyes, resulting in four groups: pHrodo-labeled CLs, pHrodo-labeled MPLCs, BCECF-labeled CLs, and BCECF-labeled MPLCs. pHrodo dye emits red fluorescence in acidic environments (pH 4–5), whereas BCECF dye emits green fluorescence under other conditions. Following intravenous administration of these dye-labeled nanoparticles into mice and subsequent euthanasia two hours later, livers from each group were extracted for fluorescence microscopic analysis. The observations revealed that CLs primarily displayed red fluorescence, indicative of endocytosis, while MPLCs exhibited predominantly green fluorescence, suggesting uptake through membrane fusion (Figure 6C). This contrast in fluorescence patterns between CLs and MPLCs implies that unlike CLs, which are primarily internalized through endocytosis, MPLCs utilize a membrane fusion pathway for cell entry.

## 3. Discussion

In this research, detailed comparative evaluations were conducted on MPLCs and CLs, focusing on their physical properties, stability, biocompatibility, delivery efficiency, and targetability. MPLCs showed a larger average diameter, lower polydispersity index, and reduced zeta potential compared to CLs. They also displayed superior membrane fusion capabilities in cancer cell lines, with significantly higher cellular uptake. Additionally, MPLCs maintained their morphology and size better than CLs when exposed to FBS, suggesting enhanced serum stability. In a xenograft mouse model using HeLa cervical cancer and ASPC pancreatic cancer cell lines, intravenous administration of MPLCs accumulated more in tumor tissues, highlighting their potential for targeted cancer therapy. Overall, these results indicate that MPLCs have superior tumor-targeting properties, possibly attributable to their membrane protein composition, offering promising prospects for enhancing drug delivery efficiency in cancer treatments.

The integration of membrane proteins into MPLCs appeared to play a pivotal role in their increased accumulation in tumor tissue. These proteins helped prevent nanoparticles from aggregating with blood serum proteins, ensuring more effective delivery to tumors. Through their role in inhibiting aggregation, MPLCs demonstrated improved stability in the bloodstream, boosting their potential to target and infiltrate tumor cells. Furthermore, the presence of membrane proteins on MPLCs may facilitate their recognition and subsequent internalization by cancer cells, as suggested by the enhanced cellular uptake observed in both HeLa cervical cancer cells and ASPC1 pancreatic cancer cells treated with MPLCs, compared to those treated with CLs. This is supported by intensified red fluorescence from the DiL dye, indicating a higher level of internalization, which was quantitatively corroborated by the analysis of fluorescence intensity values. This mechanism could be attributed to the specific interaction between the membrane proteins on MPLCs and receptors or other molecules expressed on the surface of tumor cells, potentially triggering receptor-mediated endocytosis or other uptake pathways that favor the accumulation of MPLCs within the tumor tissue. Such interactions can enhance the targeted delivery capabilities of MPLCs, making them more effective in reaching and penetrating tumor tissues compared to CLs, which lack these protein-mediated interactions.

Integrating membrane proteins into liposomes presents a promising avenue for emulating the complex functions of natural exosomes, offering an enhanced capability for cellular uptake and targeted delivery. Membrane proteins from various cell types were collectively utilized to modify liposomes to create hybrid artificial exosomes. These engineered exosomes possess evasion capabilities against phagocytosis, attributed to the abundant CD47 proteins from RBC membranes, alongside specialized tumor-homing properties conferred by cancer cell-derived proteins, including EpCAM, Galectin 3, and N Cadherin [15]. In another study, biomimetic liposomes were generated by integrating membrane proteins extracted from circulating leukocytes into lipid vesicles [12]. These leukocyte-derived proteins, including lymphocyte function-associated antigen 1, macrophage-1 antigen, and P-selectin glycoprotein ligand-1, confer cellular adhesion capabilities [13]. This unique composition enables the effective delivery of drugs like doxorubicin directly to tumor sites and surrounding tissue, an approach that surpasses the limitations often encountered with specific protein modifications.

This research utilized membrane proteins from HEK293T cells to create biocompatible artificial exosomes, referred to as MPLCs. This strategy notably advances the potential for the clinical application of these experimental findings. The HEK293T cells, a specialized derivative of the HEK293 lineage, are particularly noted for their efficiency in synthesizing proteins at a high yield, a quality that proves indispensable in the fields of gene therapy and vaccine production [18,19,20,21]. These cells are adept at proliferating in suspension cultures, even in the absence of serum, which is a cornerstone for upscaling to industrial production levels while ensuring batch-to-batch consistency. The cell line’s rapid growth rate and compatibility with various transfection techniques enhance its utility in producing a vast array of proteins [16,17]. Significantly, the human derivation of HEK293T cells ensures that the proteins undergo native post-translational modifications, which is a critical consideration for proteins intended for human therapeutic use. Given the importance of membrane proteins in the realm of pharmacology and drug development, the ability of HEK293T cells to produce such proteins not only facilitates research-grade protein synthesis but also extends to the manufacturing of biotherapeutics [20,21]. This study underscores the HEK293T cell line’s potential in advancing pharmaceutical research and commercial drug production, offering a promising avenue for the development of new therapeutic agents.

In this research, the cellular uptake mechanism of MPLCs in tumor cells was attempted to be demonstrated, showing a distinct preference for membrane fusion over the endocytic pathway commonly utilized by CLs. The MPLCs, characterized by their surface-bound positively charged membrane proteins, appear to interact attractively with the cell membrane, which inherently possesses a negative charge primarily due to phosphate groups in phospholipids and sialic acid residues in glycoproteins [22]. This electrostatic interaction is pivotal in MPLCs’ adherence and subsequent penetration into the cell membrane. It is hypothesized that the positive charge on MPLCs aids in bridging the liposomal and cellular membranes, potentially disrupting the membrane structure temporarily or, more likely, facilitating a fusion process [22,23,24,25]. This fusion, enabled by the charge interactions, allows for the direct entry of MPLCs into the tumor cells, bypassing more traditional endocytic pathways and possibly leading to more efficient intracellular delivery of therapeutic agents. This understanding of MPLC–cell membrane interactions underscores the importance of surface charge in designing effective drug delivery systems, particularly in targeting tumor cells.

In summary, this study provided an in-depth comparative analysis between MPLCs and CLs, focusing on key attributes such as size, stability, biocompatibility, and effectiveness in drug delivery. MPLCs demonstrated several advantages over CLs, including enhanced membrane fusion capabilities and higher cellular uptake in cancer cell lines. Furthermore, MPLCs showed superior stability in serum and increased accumulation in tumor tissues in xenograft mouse models, reinforcing their potential for targeted cancer therapy. The employment of membrane proteins from HEK293T cells in MPLCs, recognized for their robust productivity and manageable quality control processes, presents new possibilities for clinical applications. This aspect is particularly vital for scaling up production and maintaining consistency in therapeutic quality, adhering to cGMP standards. Therefore, the findings from this research highlight the potential of MPLCs in revolutionizing cancer treatment modalities, leveraging the unique advantages of HEK293T cells for efficient and reliable drug delivery solutions.

## 4. Materials and Methods

### 4.1. Materials

HEK293T, RAW264.7, ASPC-1, SKBR3, MCF7, and HeLa cells were obtained from Korea cell line bank (KCLB; Seoul, Republic of Korea). ASPC-1, SK-BR3, and MCF7 cells were maintained in RPMI (Hyclone, Logan, UT, USA). HEK293T, RAW264.7,1, SKBR3, and HeLa cells were maintained in DMEM/High (Hyclone). The medium was supplemented with 10% fetal bovine serum (FBS; Hyclone) and 1% penicillin–streptomycin (GibcoBRL, Carlsbad, CA, USA) at 37 °C in a humidified atmosphere with 5% CO_2_ in an incubator.

### 4.2. Preparation of Nanostructures 

The process of creating CLs involved a microfluidic technique using the NanoAssemblr Ignite system (Precision NanoSystems, Austin, TX, USA). This method allows tiny lipid particles to form through a controlled mixing of liquids. Briefly, 1,2-distearoyl-sn-glycero-3-phosphocholine (DSPC), 1,2-distearoyl-sn-glycero-3-phosphoethanolamine-N-[methoxy (polyethylene glycol)-2000] (DSPE) (Avanti Polar lipids, Alabaster, AL, USA), cholesterol (Sigma Aldrich, St. Louis, MO, USA) 3:0.15:2 molar ratio, were dissolved in organic solvent (ethanol). Subsequently, this mixture of lipids was mixed with an aqueous solution, known as phosphate-buffered saline (PBS). For generating MPLCs, membrane proteins were extracted from HEK293T cells using the Mem-PER Plus kit (Thermo Fisher Scientific, Rockford, IL, USA), and their concentration was determined using the Pierce BCA protein assay kit (Thermo Fisher Scientific). The proteins were then combined with the CLs—prepared using the same method described above—using the extrusion method. Specifically, the combination of CLs and HEK293T cell-derived membrane proteins was subjected to 15 extrusion cycles through a cellulose acetate membrane with 100 nm pores. Subsequently, both MPLCs and CLs were purified via dialysis overnight through 1000 KDa membranes (Spectrum Laboratories, Inc., Rancho, CA, USA) to eliminate unincorporated materials, enhancing their performance and stability, and were then stored at a controlled temperature of 4 °C.

### 4.3. Cell Viability Assay 

Cell viability was assessed using the Ez-Cytox assay kit (Itsbio, Seoul, Republic of Korea). Cells for immune response investigation were plated in a 96-well tissue culture plate at a density of 1 × 10^6^ cells per well in 200 µL of high-glucose DMEM medium (Hyclone), supplemented with 10% FBS (Hyclone). Following a 24 h period, 10 µL of nanoparticles were added to the cells, which were then incubated for an additional 48 h at 37 °C. Subsequently, 10 µL of EZ-Cytox reagent (Itsbio) was introduced into each well. Absorbance was measured at 450 nm using a Biotek microplate reader (Biotek, Winooski, VT, USA).

### 4.4. Nanoparticle Tracking Analysis

Nanoparticle tracking analysis was conducted using the ZetaView instrument (Particle Metrix GmbH; Ammersee, Bavaria, Germany). For each analysis, videos of 2 cycles in 11 positions were recorded. These measurements met quality standards, which included having 50 to 150 particles visible in each frame and ensuring that over 20% of the tracks were valid. The recorded videos were processed and analyzed using the ZetaView software 8.05.16.SP3 that is integrated with the instrument.

### 4.5. Cryo-TEM (Transmission Electron Microscopy) Analysis

Microscope slides (TEM grids) were prepared using a P5350/60 FEI vitrobot. A small amount (5 µL) of the sample was placed on each slide and then briefly pressed for 5 s. After waiting for 30 s, the slides were quickly frozen in liquid ethane. These frozen slides were then secured in a special holder (Gatan 626 cryo holder) for examination. The images were captured using a high-tech electron microscope (the Tecnai F20 G2; FEI company, Hillsboro, OR, USA).

### 4.6. FT-IR (Fourier Transform Infrared Spectroscopy) Analysis 

The FT-IR analysis was conducted with a spectral resolution of 0.06 cm^−1^ and 300,000:1 resolving power using a deuterated L-alanine-doped triglycine sulfate (DLATGS) detector (Leonardo Electronics, Huntsville, AL, USA). Samples were prepared by dissolving them in PBS to a final concentration of 1 mg/mL. Subsequently, 5 μL aliquots of the prepared samples were deposited onto an attenuated total reflectance (ATR) crystal. Spectral data were acquired in real time, commencing immediately upon sample application and continuing until the complete evaporation of the solvent to ensure the capture of dynamic changes in the sample composition and structure.

### 4.7. DSC (Differential Scanning Calorimetry) Analysis 

This analysis aimed to compare the thermal transitions of nanoparticles. Briefly, nanoparticles were prepared and analyzed for DSC analysis using a TG-DTA instrument (Rigaku Corporation, Tokyo, Japan). The process included putting a liquid mixture of nanoparticles in a special aluminum container. Alongside this, another aluminum container without anything in it was used for comparison. The samples were then gradually heated, starting from 28 °C and increasing to 1001 °C, at a rate of 10 °C per minute. This approach allowed for detailed observation of changes in thermal properties related to the composition of the lipid nanostructures.

### 4.8. Phagocytosis Assay

Mouse macrophage RAW264.7 cells were seeded at a density of 1 × 10^6^ cells per well in a 96-well plate. Nanoparticles were then added at a concentration of 1 × 10^7^ particles per well. After one hour of incubation, 5 µL of zymosan was added to each well, and the cells were further incubated for 3 h. Following this incubation period, the phagocytosis buffer was replaced, and the cells were imaged using a fluorescence microscope (EV OS U5000; Invitrogen, Carlsbad, CA, USA). The experimental procedure was conducted following the protocol provided by the phagocytosis kit (Abcam, Cambridge, UK).

### 4.9. Elisa

To evaluate the immunogenic response of nanoparticles in a setting that mimics in vivo conditions, nanoparticles were mixed with human red blood (Zen-bio, Durham, NC, USA) at a concentration of 250 µg/mL. The mixture was incubated at 37 °C for 2 h. After incubation, a 100 µL aliquot of the reacted blood was used to assess hemocompatibility using specific ELISA kits. The kits employed were for C5a (Invitrogen, Carlsbad, CA, USA), PF4 (RayBio, Norcross, GA, USA), CD11b (Abcam), F1 + 2 (Abbexa, Cambridge, UK), and hemoglobin (Invitrogen). The assays were conducted according to the manufacturer’s instructions provided with each ELISA kit.

### 4.10. In Vitro Tracking of Intracellular Uptake Pathway 

To elucidate intracellular nanoparticle uptake pathways in vitro, nanoparticles are labeled with a composite fluorescent probe named AS, comprising an anchor chain (A) and a signal chain (S), designed in accordance with established literature [26] and developed by Bioneer in Daejeon, Republic of Korea. The S chain incorporates a 27-nucleotide ATP aptamer sequence and is tagged at the 3′ end with a Cy3 dye for visualizing endocytic uptake routes. The A chain includes a sequence that complements the ATP aptamer and is modified at its 3′ end with cholesterol to anchor onto the nanoparticle membrane. Additionally, A is tagged at the 5′ end with a BHQ-1 dye to suppress the Cy3 fluorescence and a FAM dye within the stem to indicate membrane fusion events. In the absence of S, A can form a hairpin structure, leading to the quenching of FAM’s fluorescence by BHQ-1. During nanoparticle internalization via membrane fusion, the contents of the nanoparticle are released into the cell, while AS stays on the cell membrane surface, maintaining its original structure and causing green FAM fluorescence on the cell membrane. Conversely, in endocytic uptake of nanoparticles, AS enters the cytoplasm. Here, free ATP molecules in the cytoplasm spontaneously bind to S via the ATP aptamer, leading to the disassociation of the ATP/S complex and the subsequent revival of red Cy3 fluorescence in S, along with the self-hybridization of A and the suppression of FAM fluorescence by the BHQ-1 dye. In this research, this labeling technique offers an effective method for distinguishing between nanoparticle internalization pathways in cells, as demonstrated by the distinct green fluorescence of FAM during membrane fusion and red fluorescence of Cy3 during endocytosis. The nanoparticles were first incubated with the aptamer complex for 2 h to ensure effective tagging. Subsequently, the tagged nanoparticles were introduced to cancer cells and incubated at a constant temperature of 37 °C for a period of 24 h. To evaluate the internalization of these nanoparticles within the cells, fluorescence microscopy was utilized three hours after the treatment. 

### 4.11. Determination of Stability of Nanoparticles 

The aqueous solution of liposome and AWEsome was incubated in 0.1% FBS (diluted in PBS) for 2 h at 37 °C. Then, the samples were dialyzed overnight through 1000-kDa membranes (Spectrum Laboratories, Cincinnati, OH, USA) to remove unbounded FBS proteins. The size of purified samples was analyzed by ZetaView. The morphology of the sample was imaged by a TEM microscope (HT7800; Hitachi, Tokyo, Japan). Appropriate samples were contrasted for 4 min with 1.5% uranyl acetate. After drying, the images were acquired using a TEM microscope. To evaluate the difference in size and morphology, 0.1% FBS untreated samples were used as control. 

### 4.12. In Vitro Targetability of MPLCs

To prepare DiL-labeled nanoparticles, the nanoparticles (1 × 10^9^/mL) were incubated with 10 µg/mL DiL (1,1′-dioctadecyl-3,3,3′,3′-tetramethylindocarbocyanine perchlorate; Biotium, Fremont, CA, USA) at 37 °C for 20 min. In vitro targetability was assessed by treating 1 × 10^5^ HeLa cells and 2 × 10^5^ ASPC-1 cell lines with DiL-labeled CLs (1 × 10^7^ particles) and Dil-labeled MPLCs (1 × 10^7^ particles) for 6 h. Post-treatment, the effectiveness of targeting was verified through imaging using the EVOS M5000 Imaging System (Thermo, Waltham, MA, USA).

### 4.13. In Vivo Targetability of MPLCs

Five-week-old male BALB/c nude mice (Orient Bio, Seongnam, Republic of Korea) were used for comparative modeling of subcutaneous tumor growth. Each mouse received a subcutaneous injection of 5 × 10^6^ HeLa and ASPC-1 cells in the flank area. This study adhered to the guidelines of the Institute for Laboratory Animal Research at the Catholic University of Korea (IRB No: CUMC-2022-0317-01). To prepare DiR-labeled nanoparticles, the nanoparticles (1 × 10^9^ particles) were incubated with 10 µg/mL DiR (1,1′-dioctadecyl-3,3,3′,3′-tetramethylindotricarbocyanine iodide; Biotium, Fremont, CA, USA) at 37 °C for 20 min. For assessing in vivo targetability, mice were divided into three groups (*n* = 9 total) and received intravenous injections of PBS (control, *n* = 3), DiR-labeled CLs (1 × 10^9^ particles in 100 µL PBS, *n* = 3), or DiR-labeled MPLCs (1 × 10^9^ particles in 100 µL PBS, *n* = 3). Sequential whole-body fluorescence imaging was performed at 2 and 4 h intervals after injection using an IVIS In Vivo Imaging System (PerkinElmer, Waltham, MA, USA).

### 4.14. In Vivo Assessment of Intracellular Delivery

Five-week-old male BALB/c nude mice (Orient Bio) served as subjects for the comparative analysis of intracellular delivery mechanisms. Nanoparticles were labeled with either pHrodo or BCECF dyes (at a 20:1 dye-to-lipid molar ratio), resulting in four groups (*n* = 12): pHrodo-labeled CLs (*n* = 3), pHrodo-labeled MPLCs (*n* = 3), BCECF-labeled CLs (*n* = 3), and BCECF-labeled MPLCs (*n* = 3). These dye-labeled nanoparticles (1 × 10^9^ particles/100 μL) were intravenously administered to the mice, and two hours later, the mice were euthanized, and their livers were extracted for fluorescence microscopic analysis using the IVIS In Vivo Imaging System (PerkinElmer, Waltham, MA, USA).

### 4.15. Statistical Analysis

The data were analyzed using SPSS 11.0 software (SPSS Inc., Chicago, IL, USA). Results are presented as the mean ± standard deviation (SD). To compare groups, the Kruskal–Wallis test was used. A *p*-value of less than 0.05 was deemed significant.

## Data Availability

The datasets generated and/or analyzed during the current study are available from the corresponding author upon reasonable request.

## Supplementary Materials

The following supporting information can be downloaded at https://www.mdpi.com/article/10.3390/ijms25063294/s1.
