# Supplementary material for: Cutting-Edge HEK293T Protein-Integrated Lipid Nanostructures: Boosting Biocompatibility and Efficacy
Source: Int J Mol Sci. 2024 Mar 14;25(6):3294. doi: 10.3390/ijms25063294 (PMC10970359; doi:10.3390/ijms25063294)
Supplement: Supplementary file 1 [file ijms-25-03294-s001.zip › ijms-2867246-supplementary.pdf]

Supplementary Figure S1. Organ Distribution of CLs and MPLCs in Mouse Xenograft Models. The fluorescence of CLs and MPLCs was measured in various major organs following administration. Four hours post-injection in both HeLa (A) and ASPC1 (B) xenograft models, the liver exhibited the highest distribution of both CLs and MPLCs, followed by the spleen. A comparative analysis between the CL-treated group and the MPLCs-treated group showed that the average radiant efficiency in each organ was higher for the MPLC group than for the CL group.

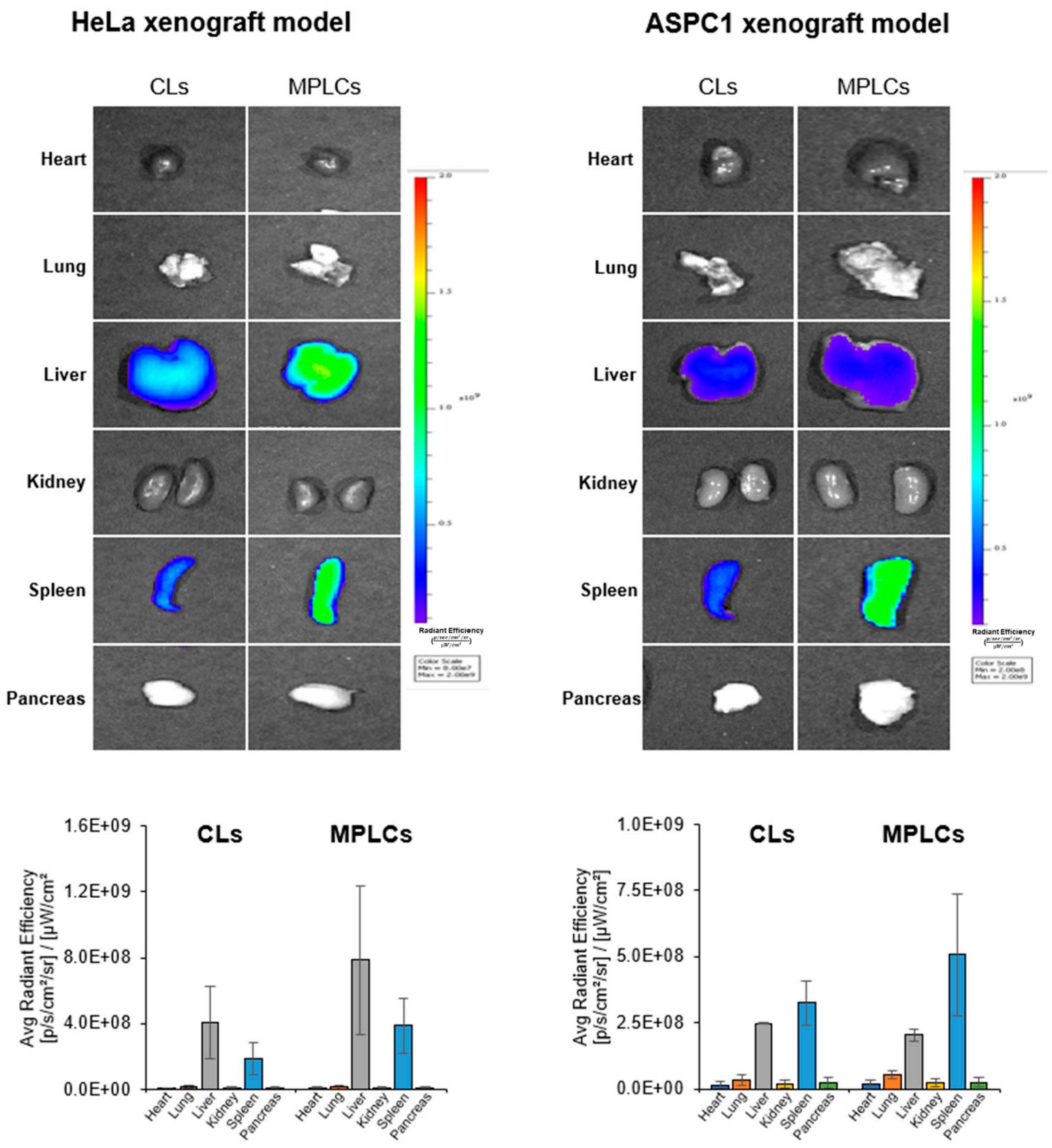
HeLa xenograft model
ASPC1 xenograft model
CLs
MPLCs
Heart
Lung
Liver
Kidney
Spleen
Pancreas
Radiant Efficiency
Avg Radiant Efficiency [p/s/cm²/sr] / [µW/cm²]
1.6E+09
1.2E+09
8.0E+08
4.0E+08
0.0E+00
1.0E+09
7.5E+08
5.0E+08
2.5E+08
0.0E+00
